# Supplementary material for: A European giant: a large spinosaurid (Dinosauria: Theropoda) from the Vectis Formation (Wealden Group, Early Cretaceous), UK
Source: PeerJ. 2022 Jun 9;10:e13543. doi: 10.7717/peerj.13543 (PMC9188774; doi:10.7717/peerj.13543)
Supplement: Supplemental Information 1 [file peerj-10-13543-s001.docx]

Supplementary information: A European giant: remains of a large spinosaurid (Dinosauria: Theropoda) from the Vectis Formation (Wealden Group, Early Cretaceous), UK.

Chris T. Barker, Jeremy A. F. Lockwood, Darren Naish, Sophie Brown, Amy Hart^5^, Ethan Tulloch, Neil J. Gostling.

# Indeterminate bone fragments:

Several other bone fragments were recovered along the stretch of foreshore near Compton Chine, covered in adhering matrix matching that of the White Rock Sandstone equivalent. Unfortunately we were unable to identify these pieces, however we provide brief descriptions and imagery (see supplementary figures file) of the larger fragments below. The other pieces are largely bone shards and not dealt with here.

## Fragment 1

Abrasion and exposure of the underling cancellous bone affects all surfaces and margins (to varying degrees), and what remains of the element has suffered three transverse cracks. As such, the true morphology of the fragment is unknown, and the element is only dealt with briefly here.

It has a maximum preserved length of 228 mm and mediolateral width of 79 mm. Exposure of the internal bone structure reveals cortical bone of various thicknesses and an absence of any internal cavitation. The cortical bone may be extremely thin in places (e.g. ~2mm where exposed in the large notch on the anterolateral surface). One side of the fragment possesses intact cortical bone and displays a flattened topology (Fig. S1A). Whilst the adjacent surface on the same side also appears similarly flattened, this portion has been abraded as evidenced by the exposure of the underlying cancellous bone. The topography of the opposite surface (Fig. S1B) is variable, producing a pair of convexities at either end; a coronal cross section through these structures would accordingly produce a hummocky margin (Fig. S1C).

## Fragment 2

The large and slightly abraded piece vaguely resembles a peduncle of an ilium, with an exposed cross-section revealing thin cortical bone. One surface of the fragment is flat (Fig. S2A), whilst the opposite produces a pair of concavities separated by a low ridge (Fig. S2B). The third preserved surface is convex and tapers towards one end (Fig. S2C). It has a maximum preserved length of 125.5 mm and height of 103.6 mm (when positioned as in Fig. S2A).

## Fragments 3 and 4

These fragments likely pertain to pieces of ilium given their triradiate (Fig. S3) and triangular cross-sections (Fig. S4), although their precise position relative to the iliac pieces described in the main text are more difficult to ascertain.

The smaller fragment of the pair has thin cortical bone and produces two largely flat sides that converge towards one another (Fig. 4A) and a notably concave third (Fig. 4B). The latter has a maximum width of 92.8 mm and is pierced by three small (and presumably vascular) foramina inset within shallow channels.

The substantially abraded surface of the larger fragment (Fig. S4A) suggesting it was closely associated with the fragments described in the main text; this abraded surface would thus correspond to the medial surface if genuine, with the opposite surface preserving part of the brevis fossa (Fig. S4B). The latter surface has a maximum width of 152.3 mm.

# Full phylogenetic results


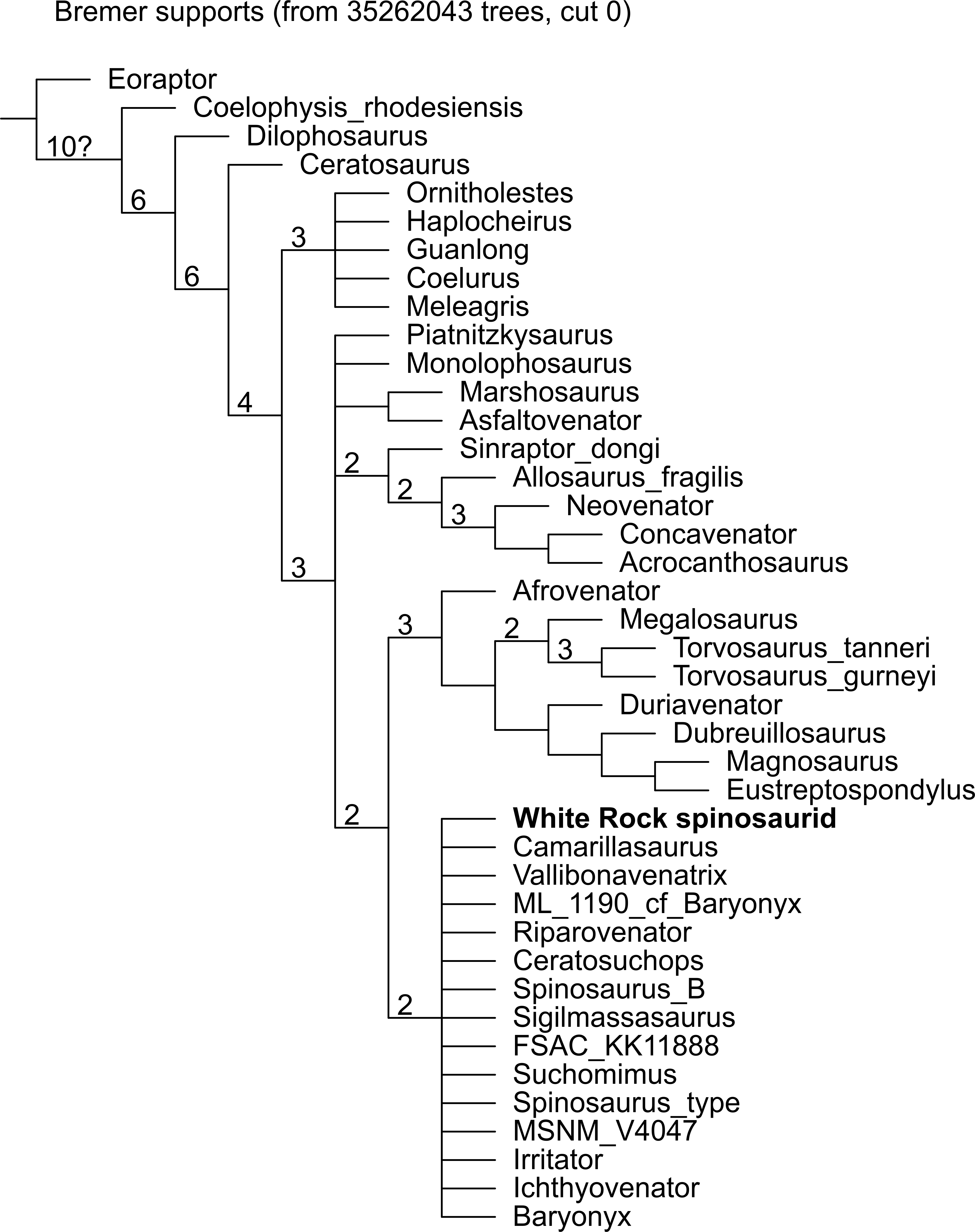


Figure S2.1 Strict consensus tree. Numbers above the nodes indicate Bremer support values (>1).


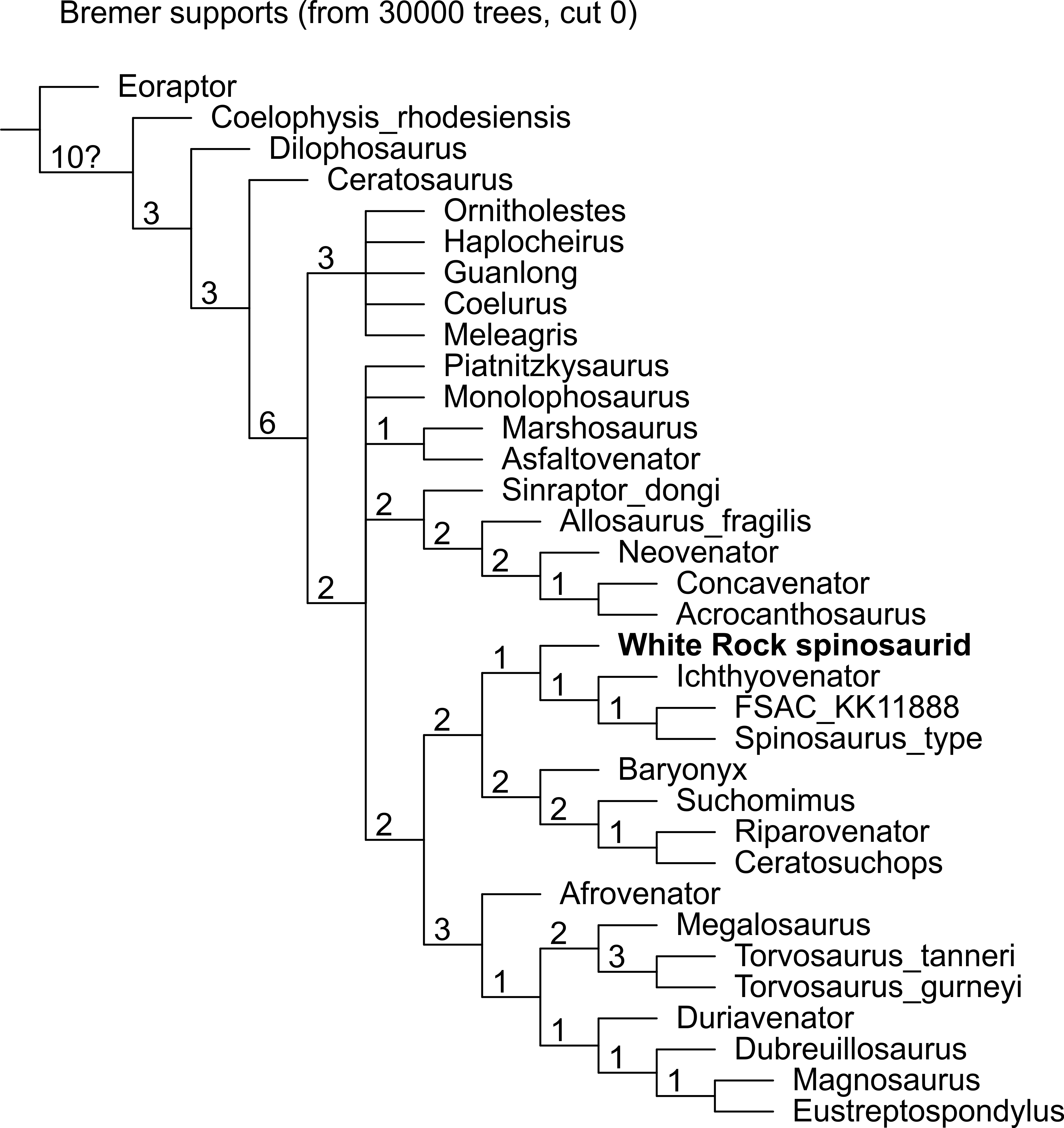


Figure S2.2 Reduced consensus following pruning of wildcard OTUs. Numbers at nodes represent Bremer support values.


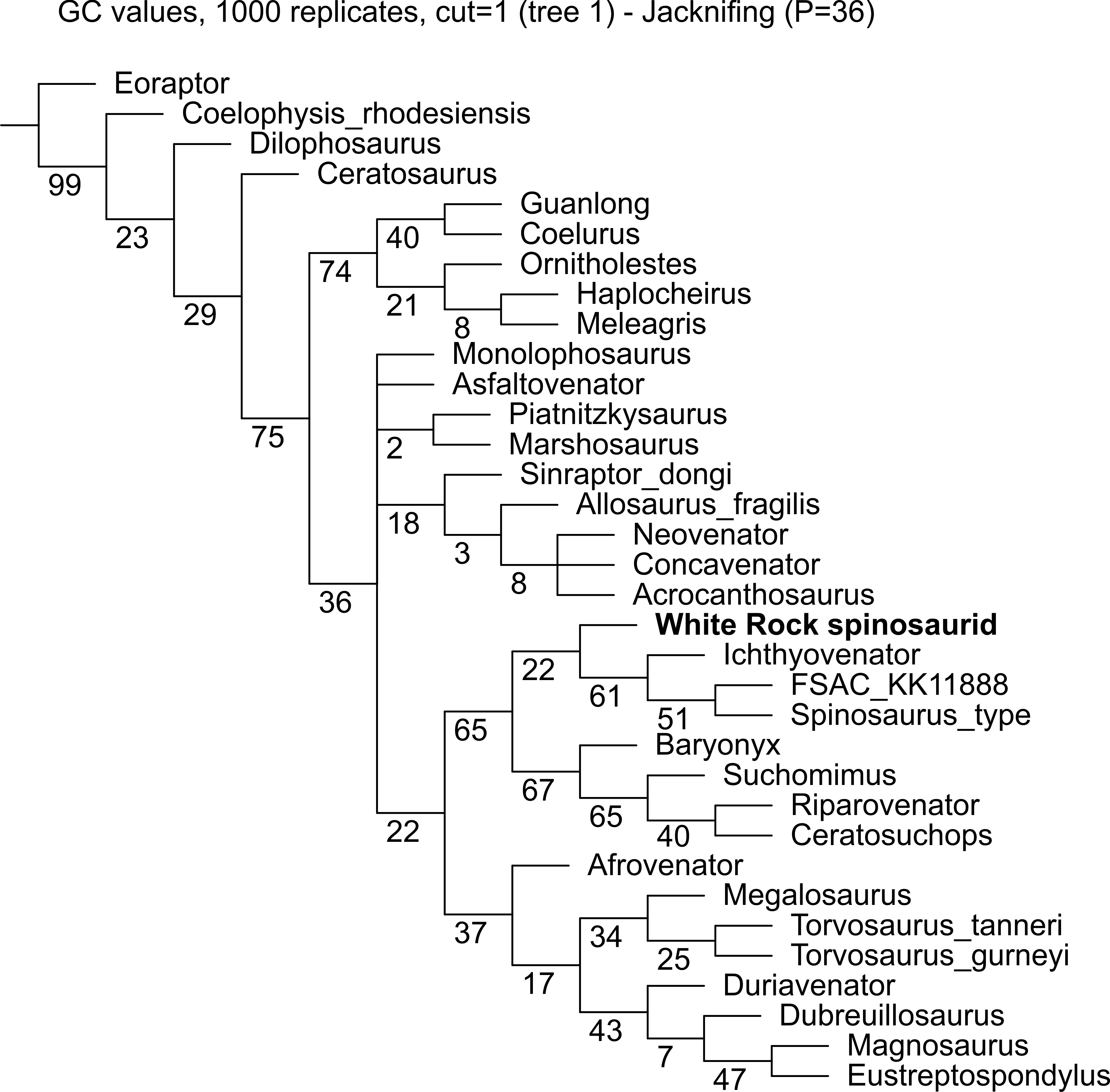


Figure S2.3 Jackknife resampling for nodal support (absolute support) following exclusion of wildcard OTUs. Numbers below nodes indicate jackknife values.


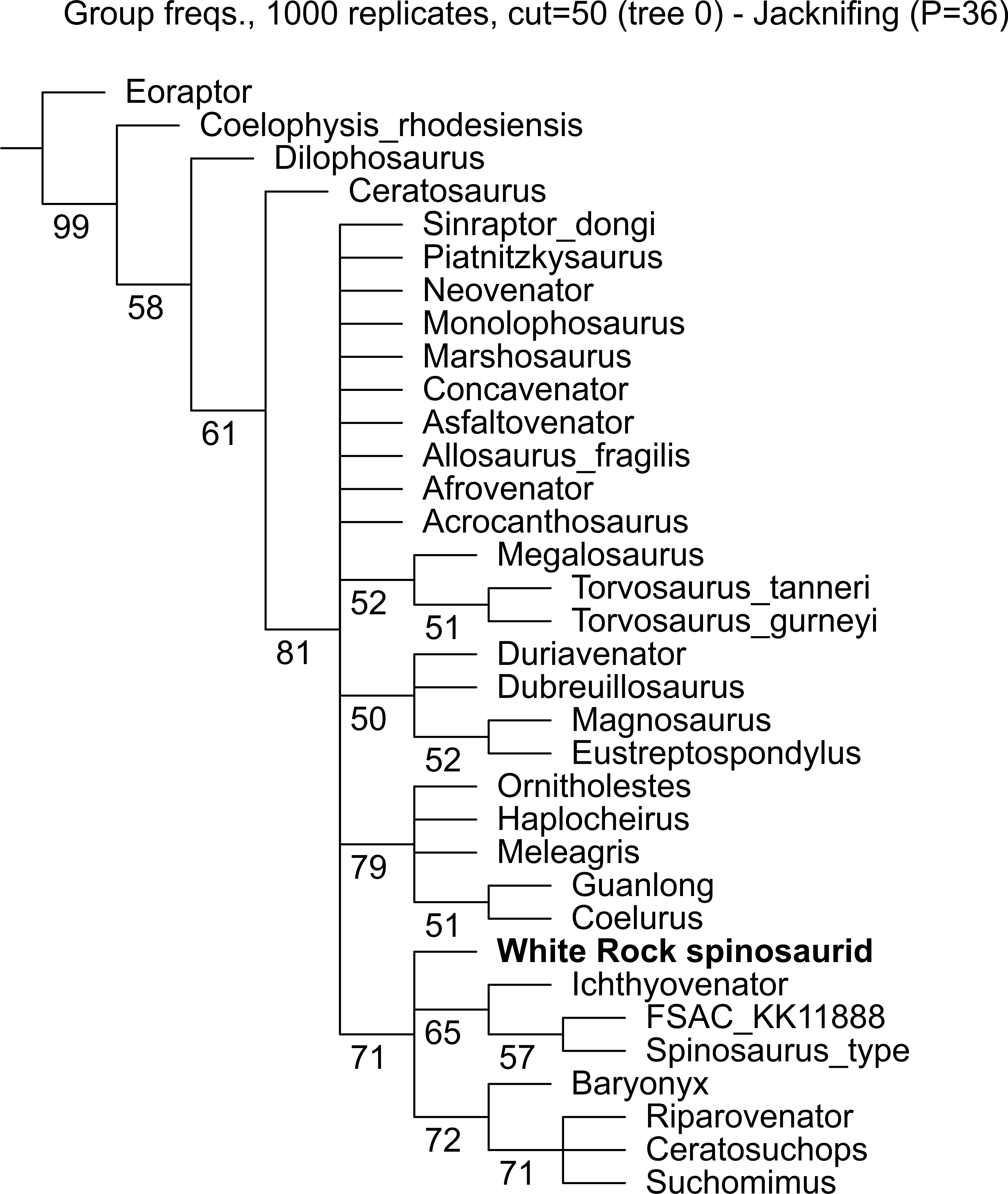


Figure S2.4 Jackknife resampling for nodal support (GC frequency values) following exclusion of wildcard OTUs. Numbers below nodes indicate jackknife values.
