# Supplementary material for: A European giant: a large spinosaurid (Dinosauria: Theropoda) from the Vectis Formation (Wealden Group, Early Cretaceous), UK
Source: PeerJ. 2022 Jun 9;10:e13543. doi: 10.7717/peerj.13543 (PMC9188774; doi:10.7717/peerj.13543)
Supplement: Supplemental Information 2 [file peerj-10-13543-s002.pdf]

Supplementary Figures: A European giant: remains of a large spinosaurid (Dinosauria: Theropoda) from the Vectis Formation (Wealden Group, Early Cretaceous), UK.

Chris T. Barker, Jeremy A. F. Lockwood, Darren Naish, Sophie Brown, Amy Hart[5], Ethan Tulloch, Neil J. Gostling.

Supplementary figure 1–4. Indeterminate bone fragments referred to IWCMS 2018.30. Dashed lines indicate surfaces presented in other views (see respective enumeration). Abbreviations: can: cancellous bone; con: concavity; cor: cortical bone; for: foramina; ri, ridge. Scale bars: 50mm.

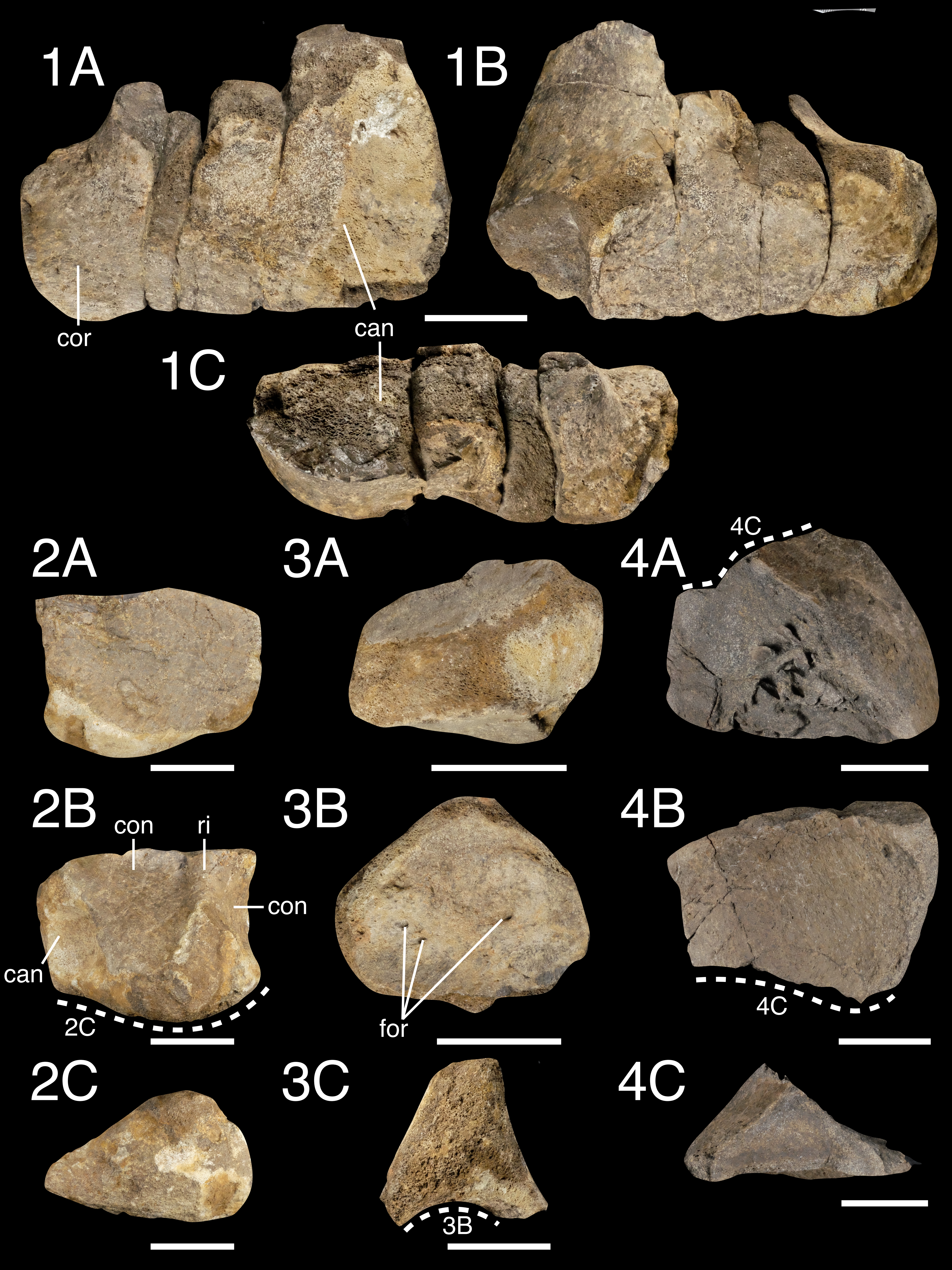
1A
1B
cor
can
1C
can
2A
3A
4A
4C
2B
con
ri
con
can
2C
3B
for
4B
4C
2C
3C
4C
3B
